# Supplementary material for: Tethering-induced destabilization and ATP-binding for tandem RRM domains of ALS-causing TDP-43 and hnRNPA1
Source: Sci Rep. 2021 Jan 13;11:1034. doi: 10.1038/s41598-020-80524-6 (PMC7806782; doi:10.1038/s41598-020-80524-6)
Supplement: Supplementary file 1 — Supplementary Figures. [file 41598_2020_80524_MOESM1_ESM.pdf]

Tethering-induced destabilization and ATP-binding for tandem RRM domains of ALS-causing TDP-43 and hnRNPA1

Mei Dang, Yifan Li and Jianxing Song*

Department of Biological Sciences, Faculty of Science; National University of Singapore; 10 Kent Ridge Crescent, Singapore 119260;

Supplementary Figures 1-6

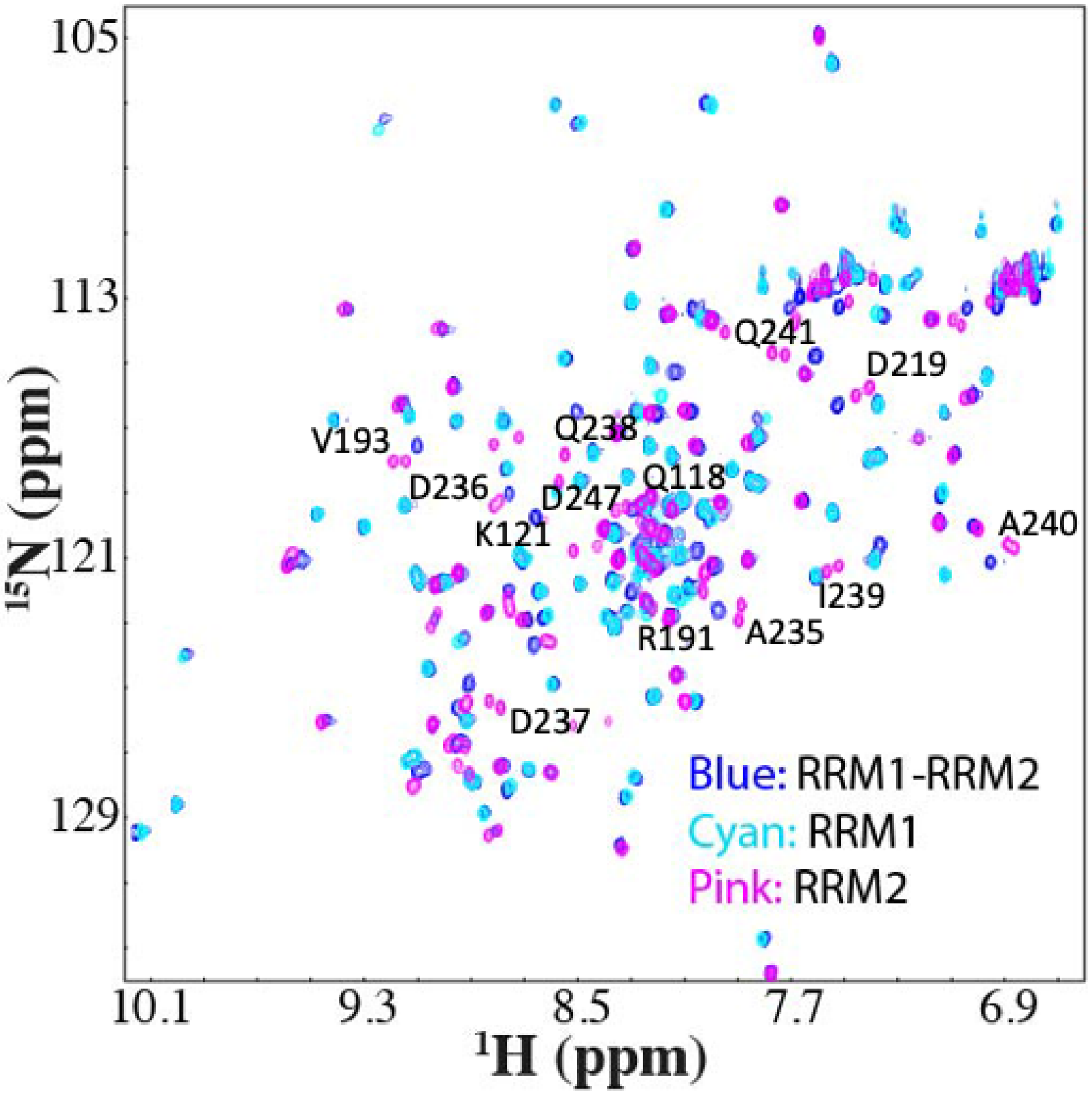

Fig. S1. Dissection-induced perturbation of TDP-43 RRM domains.
Superimposition of ^{1}H-^{15}N NMR HSQC spectra of the ^{15}N-labeled tethered RRM1-RRM2 domains (blue), the isolated RRM1 (cyan) and RRM2 (pink) proteins with the significantly shifted HSQC peaks labeled.

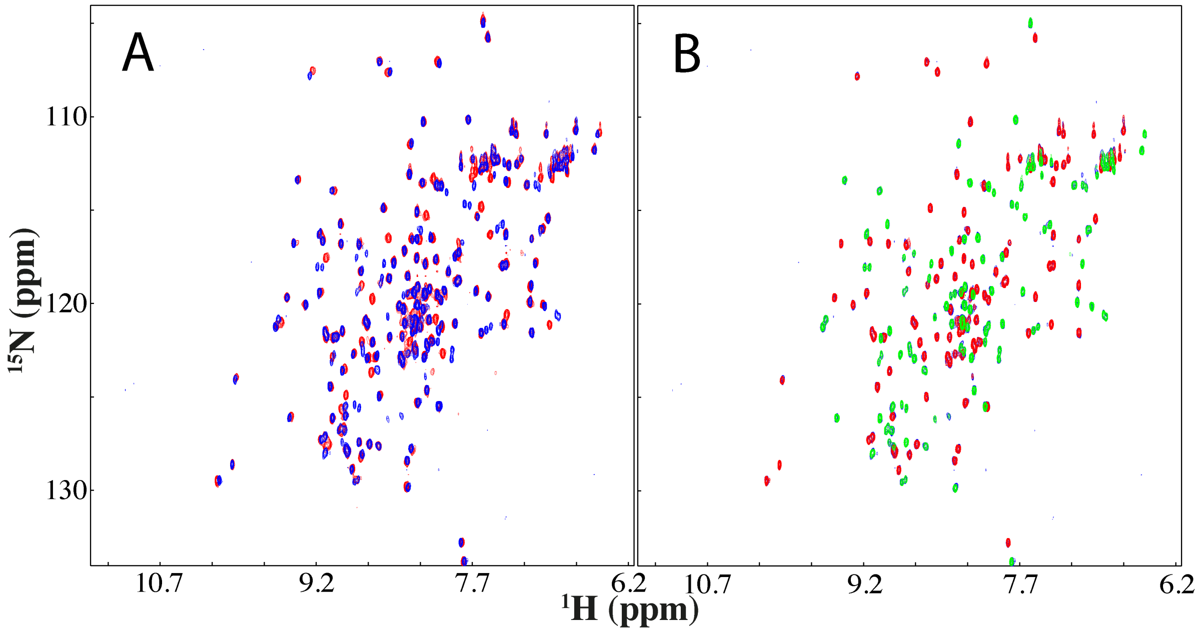

Fig. S2. Inter-domain interaction of TDP-43 RRM domains.
(A) Superimposition of HSQC spectra of the ^{15}N-labeled tethered RRM1-RRM2 domains (blue), and the mixture of the isolated RRM1 and RRM2 at 1:1 (red). (B) Superimposition of HSQC spectra of the mixture of the isolated RRM1 and RRM2 at 1:1 (blue) and the isolated RRM1 (cyan) and RRM2 (red).

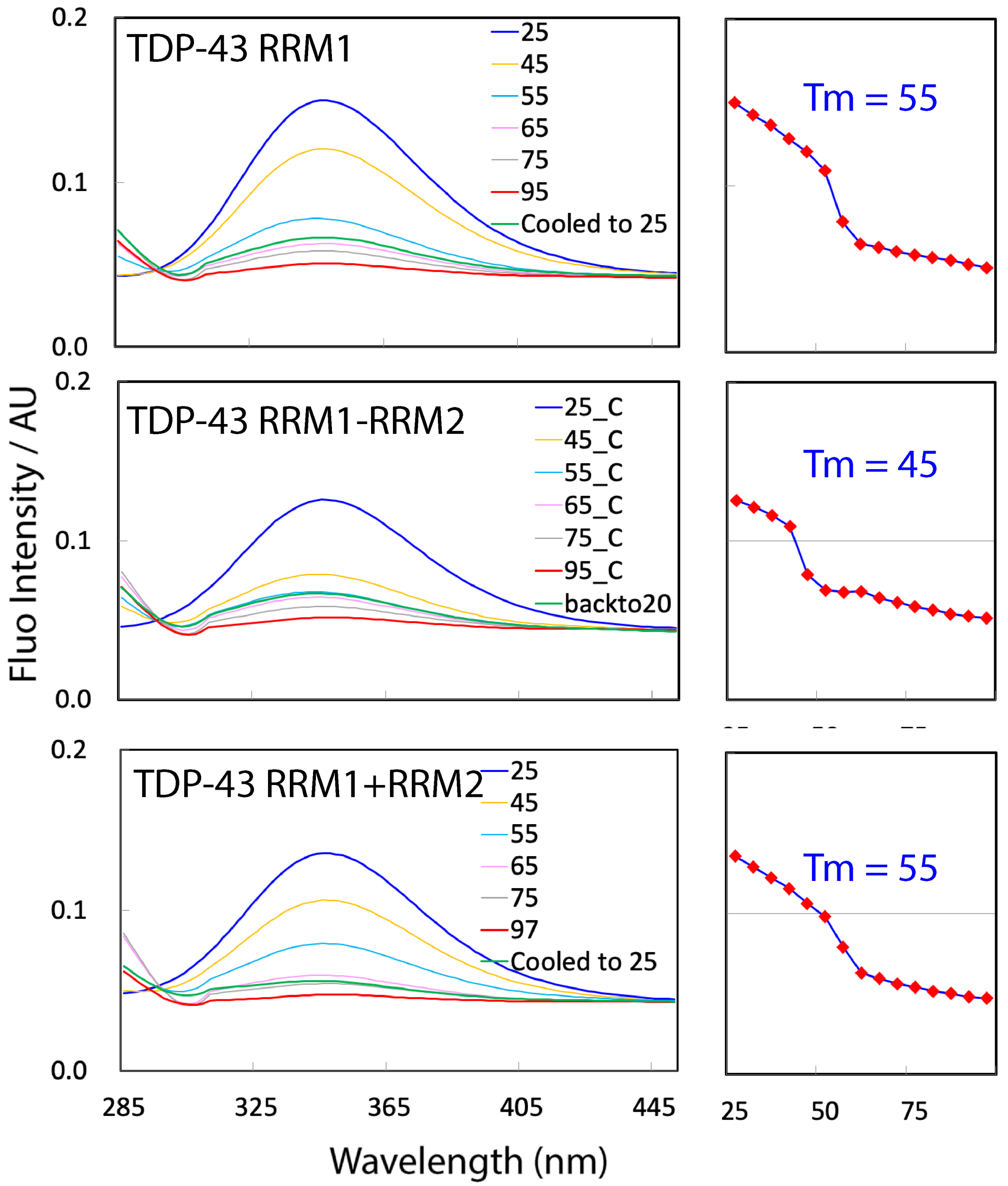

Fig. S3. Thermal denaturation of TDP-43 RRM domains by fluorescence spectroscopy. Spectra of intrinsic Trp UV fluorescence and denaturation curves reported at 347 nm of TDP-43 RRM domains in the different forms with temperatures ranging from 25 to 95 °C. The fluorescence intensity was reported in arbitrary unit.

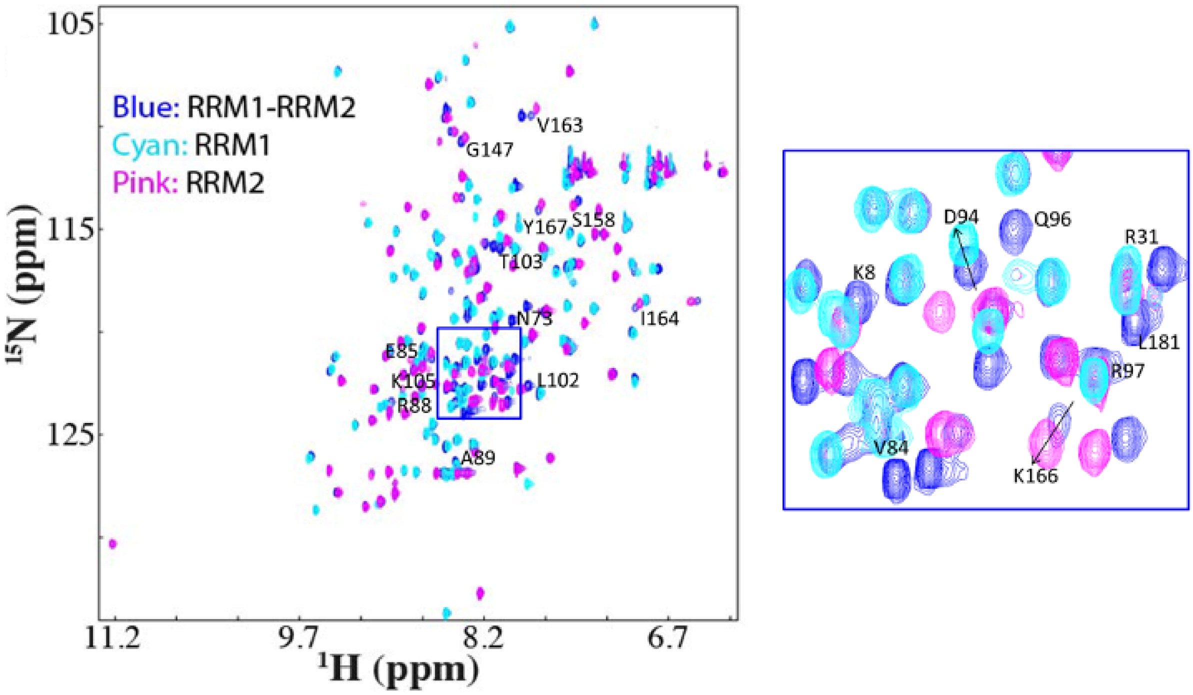

Fig. S4. Dissection-induced perturbation of hnRNPA1 RRM domains.
Superimposition of ^{1}H-^{15}N NMR HSQC spectra of the ^{15}N-labeled tethered RRM1-RRM2 domains (blue), the isolated RRM1 (cyan) and RRM2 (pink) proteins with the significantly shifted HSQC peaks labeled.

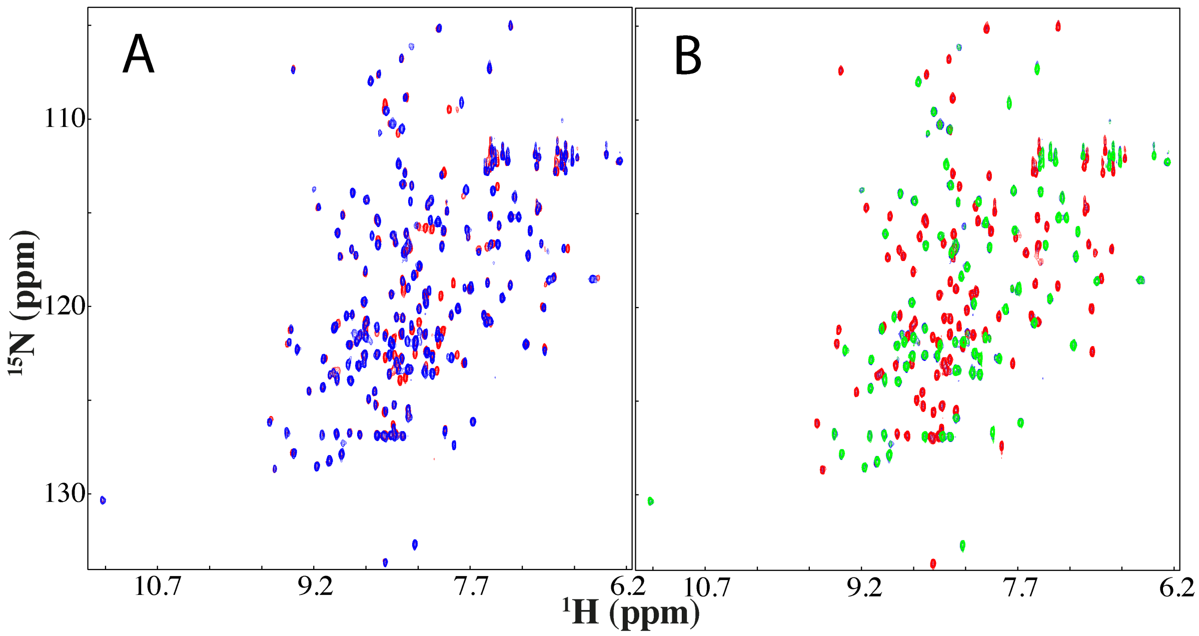

Fig. S5. Inter-domain interaction of hnRNPA1 RRM domains.
(A) Superimposition of HSQC spectra of the ^{15}N-labeled tethered RRM1-RRM2 domains (blue), and the mixture of the isolated RRM1 and RRM2 at 1:1 (red). (B) Superimposition of HSQC spectra of the mixture of the isolated RRM1 and RRM2 at 1:1 (blue) and the isolated RRM1 (cyan) and RRM2 (red).

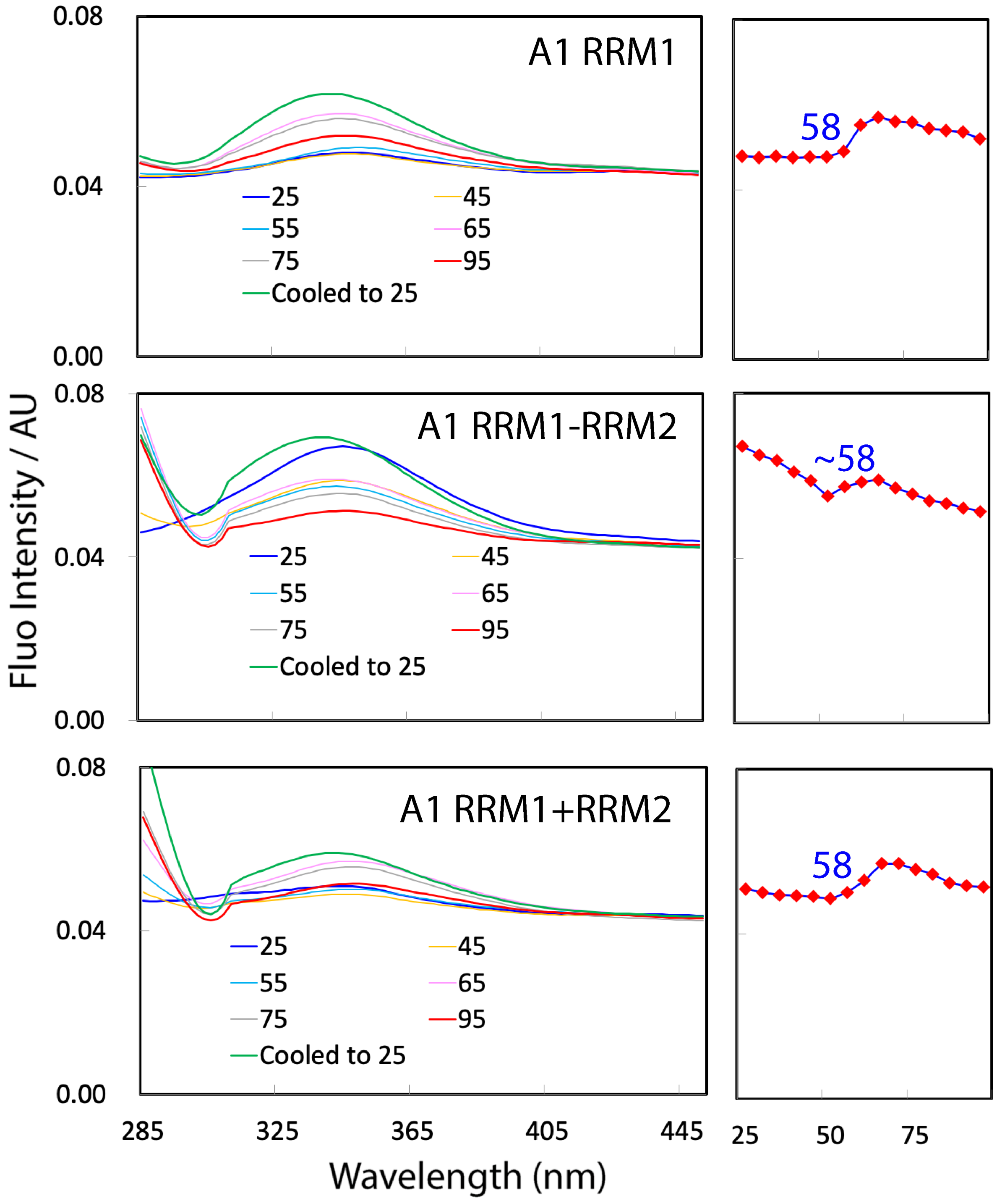

Fig. S6. Thermal denaturation of hnRNPA1 RRM domains by fluorescence spectroscopy. Spectra of intrinsic Trp UV fluorescence and denaturation curves reported at 347 nm of hnRNPA1 RRM domains in the different forms with temperatures ranging from 25 to 95 °C. The fluorescence intensity was reported in arbitrary unit.
